# Bioclimatic dataset of Metropolitan France under current conditions derived from the WorldClim model

**DOI:** 10.1016/j.dib.2020.105815

**Published:** 2020-06-04

**Authors:** Gwenhael Perrin, Sébastien Rapinel, Laurence Hubert-Moy, Frédéric Bioret

**Affiliations:** a Univ Brest, EA 7462 Géoarchitecture, F-29200 Brest, France; b University of Rennes, LETG, UMR 6554 CNRS, place du recteur Henri Le Moal, 35000 Rennes, France

**Keywords:** Bioclimatology, ecology, Worldwide Bioclimatic Classification System, spatial data, bioclimatic variables

## Abstract

Several studies have shown that adequate bioclimatic information is of major importance for mapping ecological niches or for modelling the distribution ranges of species and communities, particularly from a climate change perspective [1,2]. However, in France, there are few data sources that provide consistent information, available data being produced at low spatial resolution and based on classification systems that are not suitable for mapping French ecological systems. This paper presents bioclimatic maps produced on Metropolitan France and based on the Worldwide Bioclimatic Classification System, which are called Global Bioclimatics [3].

This data paper documents a set of variables that includes 23 bioclimatic maps generated according to the Worldwide Bioclimatic Classification System. These maps describe current bioclimatic conditions in Metropolitan France at a resolution of 30 arc-seconds. Climatic parameters and bioclimatic indices usually used for the analysis or modelling of species and communities’ distribution, and bioclimatic typological units, were calculated using the temperature and precipitation data derived from the WorldClim 2 model. These maps can be used in GIS or models by researchers for mapping ecological conditions, but can also provide natural resource managers with analytical tools to assess Nature conservation policies.

**Table:** 

Subject	Agricultural and Biological Sciences (General); Environmental Science (General)
Specific subject area	Bioclimatology
Type of data	Spatial raster dataset (GeoTiff)
How data was acquired	Data were extracted from WorldClim Global Climate Data platform (http://worldclim.org/data/worldclim21.html)
Data format	Processed; Descriptive
Parameters for data collection	Average, minimum and maximum temperatures and precipitation data were obtained from WorldClim v2.1 (30 arc-seconds, i.e. about 0.6 km^2^ spatial resolution; WGS-84 projection system (EPSG: 4326); period: 1970-2000). A mask was applied to extract these climatic variables for Metropolitan France.
Description of data collection	Bioclimatic variables were generated based on the Worldwide Bioclimatic Classification System [3]. Four temperature and precipitation parameters were calculated to derive eleven bioclimatic indices. These parameters and indices were used to provide eight maps of bioclimatic typological units (bioclimates, thermotypic horizons, ombric types...).
Parameter and index acronyms and definition of bioclimatic units are provided in Rivas-Martínez *et al.*[3].	
Data source location	France
Data accessibility	With the article

Value of the Data

• The dataset provides bioclimatic maps that can be used in GIS or models for mapping ecological conditions
• The dataset can be of interest in many fields of research (Ecology, Biogeography, Forestry, Agronomy…) to build models of species, plant communities or vegetation series distribution
• The generated maps can also provide natural resource managers with analytical tools to assess Nature conservation policies or agricultural practices

## Data

Several studies have shown that adequate bioclimatic information is of major importance for mapping ecological niches or for modelling the distribution ranges of species and communities, particularly from a climate change perspective [1,2]. However, in France, there are few data sources that provide consistent information, available data being produced at low spatial resolution and based on classification systems that are not suitable for mapping French ecological systems. This paper presents biocimatic maps produced on Metropolitan France and based on the Worldwide Bioclimatic Classification System, which are called Global Bioclimatics [3].

The Worldwide Bioclimatic Classification System offers a quantifiable bioclimatic typology that shows a close relationship between climate and vegetation models [4]. It recognizes 28 bioclimates and about 400 isobioclimates (i.e. aggregation of bioclimates, thermotypic horizons and ombric horizons), highlighting slight climatic variations. Compared to other widely used bioclimatic classification systems such as that of Köppen [5], the Worldwide Bioclimatic Classification System is adapted to the context of the French ecosystems: it discriminates between subtropical and mediterranean climates and considers mountains belts like altitudinal thermic variations of the global surrounding bioclimate – *i.e.* as a part of the zonation going from low to high altitudes – rather than like a distinct orobioclimate. Moreover, it includes several levels of submediterraneity (*Ios_i_* and *Isbm* indices in Table 1), which is an important factor in temperate ecosystems of southwest Europe where Mediterraneo-Atlantic species are found.

**Table 1:** List of the generated maps and associated range of values. Me. for Mediterranean macrobioclimate (n=53867 px); Te. for Temperate macrobioclimate (n=874226 px)

*Type of data*	*Layer*	*Range of values*	
**Parameters**	Annual positive temperature in tenths of°C (*Tp*)	Me. Te.	907 – 2070 2 – 1875
	Annual negative temperature in tenths of°C (*Tn*)	Me. Te.	0 – 0 -1013 – 0
	Average temperature of the summer quarter in tenths of°C (*Ts*)	Me. Te.	453 – 746 -64 – 697
	Annual positive precipitation in mm (*Pp*)	Me. Te.	463 – 1023 44 – 1651
**Indices**	Simple continentality index in°C (*Ic*)	Me. Te.	11.9 – 19.4 7.2 – 19.4
	Diurnality index in°C (*Id*)	Me. Te.	3.9 – 14.3 3.0 – 14.4
	Annual ombrothermic index (*Io*)	Me. Te.	2.29 – 9.96 4.08 – 240.00
	Annual ombro-evaporation index (*Ioe*)	Me. Te.	0.56 – 50.51 0.83 – 180.48
	Monthly estival ombrothermic index (*Ios1*)	Me. Te.	0.27 – 2.27 0.83 – 47.86
	Bimonthly estival ombrothermic index (*Ios2*)	Me. Te.	0.47 – 1.80 1.34 – 240.00
	Trimonthly estival ombrothermic index (*Ios3*=*Iosc3*)	Me. Te.	0.53 – 2.16 1.80 – 240.00
	Fourmonthly estival ombrothermic index (*Ios4*=*Iosc4*)	Me. Te.	0.71 – 2.73 2.24 – 240.00
	Submediterraneity index (*Isbm*)	Me. Te.	- 0 – 471
	Thermicity index in tenths of°C (*It*)	Me. Te.	91 – 394 -383 – 354
	Compensated thermicity index in tenths of°C (*Itc*)	Me. Te.	91 – 394 -383 – 354
**Bioclimatic typological units**	Bioclimates	Me. Te.	Only pluvioseasonal oceanic Oceanic to hyperoceanic
	Bioclimatic variants	Me. Te.	Only normal Normal-steppic-submediterranean.
	Continentality levels	Me. Te.	Strong euoceanic to strong semicontinental Weak euhyperoceanic to strong semicontinental
	Isobioclimates	Me. Te.	See ranges of the constitutive variables
	Macrobioclimates	-	Mediterranean and temperate
	Ombric horizons	Me. Te.	Lower dry to upper humid Lower subhumid to ultrahyperhumid
	Submediterraneity levels	Te.	Weak submediterranean to highly strong submediterranean
	Thermotypic horizons	Me. Te.	Upper thermomediterranean to upper supramediterranean Lower thermotemperate to upper cryorotemperate

The WorldClim 2 model [6] was used for the current period (1970-2000) to derive the entire dataset. The overall model accuracy is considered by its authors to be very high for temperatures, while precipitation modelling is a bit poorer due to a more heterogeneous regime in time and space than that of temperatures.

Bioclimatic maps that cover Metropolitan France were generated with the original coordinate reference system (WGS-84) at the resolution of 30 arc-seconds (*i.e.* about 0.6 km^2^).

The dataset contains 23 maps, including 4 climatic parameters, 11 bioclimatic indices and 8 bioclimatic typological units (Table 1).

**Figure 1:**
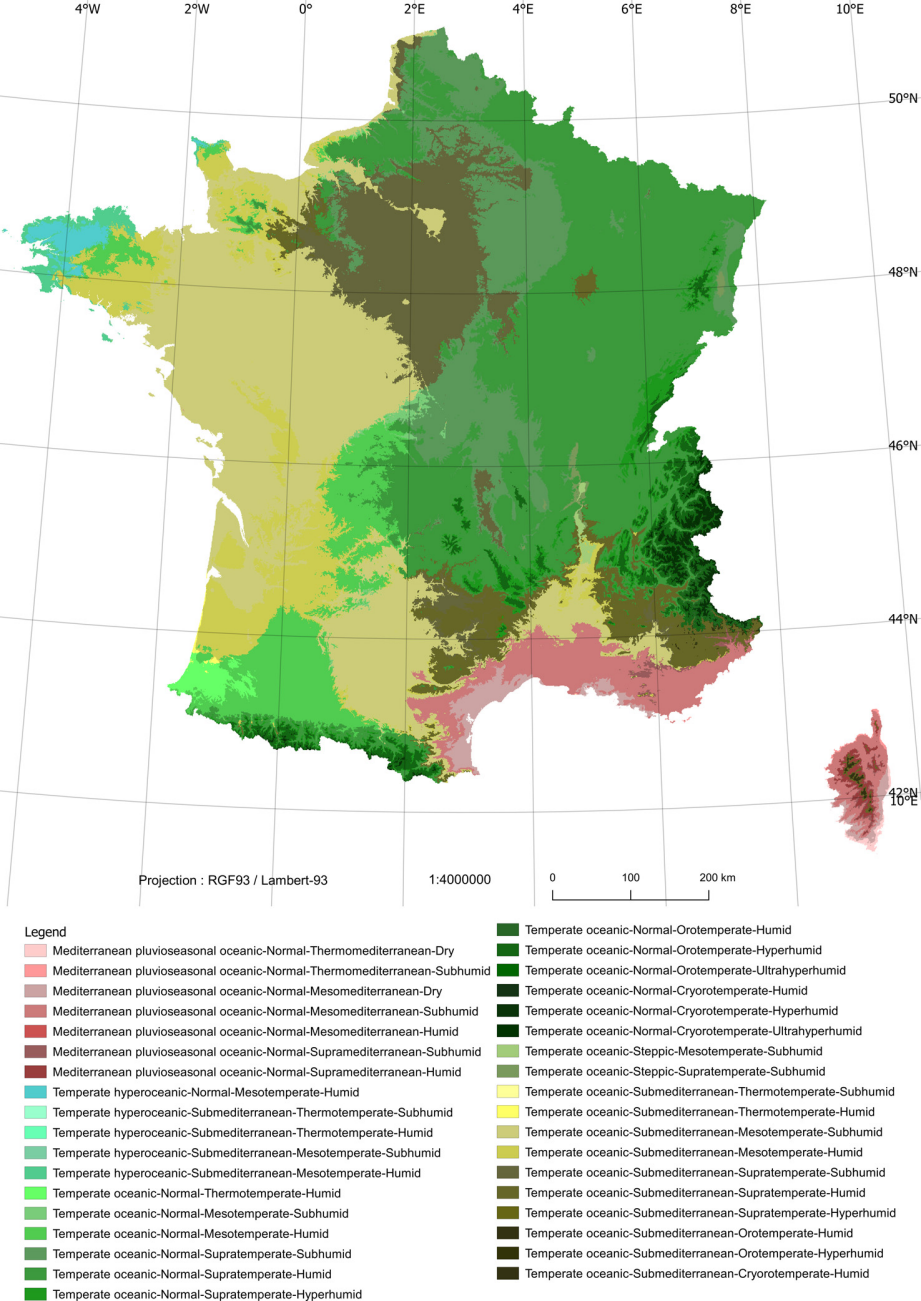
Example of a bioclimatic map that covers Metropolitan France generated with the WorldClim Global Climate Data for the 1970-2000 period: isobioclimates including bioclimatic variants

An overview of the results is given in Fig. 1.

## Experimental design, materials and methods

The bioclimatic dataset was derived from the WorldClim v2.1 dataset [6]. Temperature (*i.e.* annual average, minimum and maximum temperatures) and annual amount of precipitation variables were obtained at a spatial resolution of 30 arc-seconds. The digital elevation model with a grid resolution of 250m (source: BD ALTI® IGN, French Geographic National Institute), which was used to calculate some of the bioclimatic variables, was aggregated to a 30 arc-seconds spatial resolution.

The procedure for calculating bioclimatic variables (*i.e.* generation of the climatic parameters and bioclimatic indices first and then the bioclimatic units) was first carried out as defined by [3,7]. Then, following the recommendations of the authors, some of these bioclimatic variables were compensated. For example, temperatures variables (*i.e. T, M, m, Itc* and *Tp*) were corrected by the altitude to produce maps of the macrobioclimates (see note 1 of table 25 in [3]).

Data processing was performed using two softwares [8]. Most bioclimatic variables were calculated using simple arithmetic operations or conditional statements with QGIS 3. The other bioclimatic variables were calculated with the raster functions of GRASS: r.latlong for latitude definition; r.slope.aspect for topographic parameters; r.series for statistic operations; r.reclass for segmentation; r.sun to determine the day length (*L* parameter) of the Thornthwaite equation [9] that is required for the annual ombro-evaporation index (*Ioe*) [7]. Calculation of the day length was made on the median day of each month and the *step* parameter was set to 0.025.

Appendix A. Supplementary data

## Declaration of Competing interest

The authors declare that they have no known competing financial interests or personal relationships that could have appeared to influence the work reported in this paper.

## Appendix Supplementary materials

Image, application 1
