# Supplementary material for: Bioclimatic dataset of Metropolitan France under current conditions derived from the WorldClim model
Source: Data Brief. 2020 Jun 4;31:105815. doi: 10.1016/j.dib.2020.105815 (PMC7300140; doi:10.1016/j.dib.2020.105815)
Supplement: Supplementary file 1 [file mmc1.zip › bioclimatic_maps/typological_units/_Codification of the bioclimatic typological units maps.pdf]

Codification of the bioclimatic typological units maps

Macrobioclimate

Unit	*Code*	*Acronym*
**Mediterranean**	1	Me
**Temperate**	2	Te

Bioclimate

Unit	*Code*	*Acronym*
**Mediterranean pluvioseasonal oceanic**	11	Mepo
**Temperate hyperoceanic**	21	Teho
**Temperate euoceanic**	22	Teoc

Bioclimatic variants

Unit	*Code*	*Acronym*
**Normal**	0	Nor
**Steppic**	1	Stp
**Submediterranean**	2	Sbm

Thermotypic horizons

Unit	*Code*	*Acronym*
**Lower thermo-**	11	T
**Upper thermo-**	12	T
**Lower meso-**	21	M
**Upper meso-**	22	M
**Lower supra-**	31	S
**Upper supra-**	32	S
**Lower oro-**	41	O
**Upper oro-**	42	O
**Lower cryoro-**	51	C
**Upper cryoro-**	52	C

Ombric horizons

Unit	*Code*	*Acronym*
**Lower dry**	11	Dry
**Upper dry**	12	Dry
**Lower subhumid**	21	Shu
**Upper subhumid**	22	Shu
**Lower humid**	31	Hum
**Upper humid**	32	Hum
**Lower hyperhumid**	41	Hhu
**Upper hyperhumid**	42	Hhu
**Ultrahyperhumid**	51	Uhh

Continentality levels

Unit	*Code*
**Weak euhyperoceanic**	12
**Strong subhyperoceanic**	21
**Weak subhyperoceanic**	22
**Strong semihyperoceanic**	31
**Weak semihyperoceanic**	32
**Strong euoceanic**	41
**Weak euoceanic**	42
**Weak semicontinental**	51
**Strong semicontinental**	52

Submediterraneity levels

Unit	*Code*
**Extremely weak submediterranean**	11
**Highly weak submediterranean**	12
**Weak submediterranean**	13
**Strong submediterranean**	21
**Highly strong submediterranean**	22

The codification of some layers such as bioclimatic variants, thermotypic horizons and isobioclimates results from the concatenation of the codes of the layers that constitute them.
